# Letter from Birmingham

**Published:** 1890-10

**Authors:** Henry K. Leake

**Affiliations:** Birmingham, England


### LETTER FROM BIRMINGHAM:—TAIT'S OPERATIONS, ETC.

*Editor Daniel's Texas Medical Journal:*

The last operation performed by Tait to-day was one for vesico-vaginal fistula. The woman was placed on her side, Sims' speculum introduced, milk injected into the bladder and which, running through into the vagina, discovered the location and size of the fistula. It was situated about half way the anterior wall of the vagina. Tait was kneeling upon a low foot stool. He seized the margin of the opening with a long handled tenaculum, raised the tissues and with a narrow bladed bistoury rapidly cut out the fistula by including it in the elliptical piece excised. With his peculiarly shaped needle, three or four silver wire sutures were now inserted, which were twisted by the ends of his fingers, a Sigmoid catheter introduced into the bladder and the operation was complete. Simplicity and rapidity here, as in everything he does, was "the order of the day."

Several years ago, at Dallas, a woman applied to me with evidences of suppuration in the iliac region, which was considered to be an abscess formed, or forming, in the vicinity of the ovary. A long and deep incision was made, when exit was given to a large quantity of offensive pus, after which the cavity was thoroughly washed out and drained. Healing was slow and never complete, for a sinus was left through which, for many months, menstruation regularly recurred; thus demonstrating a connection with the Fallopian tube. The case passed out of my hands as I refrained from trying further operative measures. A similar case was seen by Tait on yesterday, its pathology believed to be the same as the one just narrated, and an operation decided on. Determining the direction of the sinus with a probe, the abdominal cavity was opened low down, the end of the incision terminating well into the tissue of the mons. Tait said that an old adherent ovary and tube were the cause of the trouble. These organs were imbedded in a mass of dense, well organized exudate involving the abnominal wall, uterus, bladder and broad ligaments. An attempt was made to free and raise them to the abdominal incision—a task which sorely tried even the skill and perseverance of Tait. Necessarily, there was much traumatism, and this involved a considerable rent in the bladder, but extirpation of the diseased parts was ultimately accomplished. The hole in the bladder was promptly sewn up, irrigation made, and a drainage tube introduced. A Sims' catheter was left in the bladder. Tait remarked that a man might well hesitate to operate in such cases, and that he would express no opinion concerning the future of the patient just operated on. However, twenty-four afterwards the case is progressing finely.

More than two weeks have now elapsed since Tait performed a Porro operation after his method, which modifies the original procedure, and which I have elsewheıe described. Yesterday morning, Tait exhibited the patient to the class. You may partly conceive how deeply impressed we were with the wonderful resources of our art when utilized by a master hand, as we stood at the bedside of this patient who, smiling with maternal pride and contentment, fondled her lusty young Briton as though she had never passed through the serious ordeal which gave him to her. And when Tait approached the bed, the very climax of gratitude and reverence was expressed in every line of her face. I thought that Tait, himself, was strongly moved, inwardly, although he said not a word which betrayed any emotion of satisfaction. He vouchsafed but one remark:—"Gentlemen, there has been no rise of temperature of consequence."

On yesterday after the day's work was over, Tait said:—"You all dine with me at half past seven o'clock;" and so we did, only the members of the class being present. To thoroughly know this remarkable man, you must meet him on occasions disassociated from his professional work, or at least, as far as this is possible, for wherever he may be, he is more or less constantly reminded of his patients by messages—verbal, written and telegraphic. On the occasion referred to, we formed an enjoyable symposium, with Tait in the triple role of genial host and proposer and umpire of our conversation—a position to which he was in all respects entitled although, much unlike the vain Coleridge, Tait arrogates not to himself all the talking. To give you even a synopsis of the subjects discussed—we rose from the table at 11 o'clock p. m.—I shall not attempt, but I may say that Tait somewhat unburdened his soul on the question of antiseptics—a thing in which he takes the greatest interest, and argus-eyed, watches its different and developing phases. Tait is a thorough believer in the existence and true influence of germs—he has often seen and studied them. He knows that they manufacture products which may, or may not, be poisonous. He knows also, that they are the veritable scavengers of the human system as of that of other animals. They dispose of dead animal tissue; they will not, they cannot—*rei natura*—attack or in any way injure that which is vitally active. The latter is unfruitful soil—it repels them. This is the ordering of nature—a harmonious working out of means to ends, and the ends specifically justify the means; but if this be the *naturæ lex* and undesirable, or rather disastrous in its immediate or remote effect then the *artis lex* may supplant the entire process by limiting severely the function of the germ—there must be no ends to meet, or as little as possible. So is the life history of the germ—its function—understood; a fact established not so much by cultures or microscopical investigations which continually befog us in theoretical tergiversations that we vainly expect to yield practical results in surgery, but by closely observing the conditions under which germs thrive and multiply, and without regard to their essential form and movement. It is time he says—speaking broadly—that we largely return to the teaching of the fathers of medicine while not undervaluing much that modern times have demonstrated, or at least, may foreshadow. Let us study more the natural history of disease, and let us do this more intelligently, more systematically. But as regards the case in question—suppuration—this is a disease! Its essential nature we may not understand despite the many theories and dimly outlined facts advanced in this direction. As in the case of the French nobles who spurned the theories of Rousseau—if I recollect aright—the theories we reject may yet return and "flog" us, but the "flogging" will be none the less hard to bear if we are true to our convictions now. "Every action is consistent so it be honest in its hour." True! but it is very questionable in medicine at least, how much of honest, or rather of common sense and practical result there is in being blown about "by every wind of doctrine." Germs may have much to do with suppuration, but we have grasped enough of the governing fact to show that devitalization of tissue is, for us practically, to be considered the *fons et origo* of the diseased process. Here is the basal fact, the established proposition, so to speak; the germ is the corrollary—the natural concomitant, even the welcome arrival upon the scene in some cases. But we look apart from the germ to account for this devitalization. Does not this devitalization often occur deep within the tissues of the body? How does suppuration begin in the hip joint? How are deeply situated cold abscesses formed? Admitting the restless, aggressive and ubiquitous nature of the germ can it, like the screwworm or the trichina, bore an entrance to such remotely situated regions? But if these microscopic harpies are carried by all the many devious channels of the body—arteries, veins and lymphatics—why are not our systems a continual riddle of more or less large abscess cavities? Why is pus an adventitious, a hurtful, nay! a fatal component of the animal economy? The differences in the form and nature of the germ will not here supply the answer, for if the pyogenes aurcus and the streptococcus be said to be the specific cause of the suppuration in deep abscess, as well of that on open surfaces, were they not in the former case circulating in the healthy body; or did they exceptionally and instinctively bore an entrance for the express occasion? How were they intelligently directed to certain points? If they normally exist in the human body, why do they not oftener cause suppuration? On the other hand, is it not rather the condition of the soil which attracts and develops the germ? But if the farmer properly prepares and keeps clear the ground how can the tares grow? If the surgeon makes the wound with the minimum amount of traumatism, and refuse materials be given full and speedy exit, how can the germ subsist with such unnatural environments? As a matter of fact, shown by Tait's results, do they so subsist and multiply, or do they die of inanition? Shall we look to the soil, therefore, and not to the germ as the prime consideration? In other homely words, but none the less expressive, shall we begin "at the right, or the wrong end of the stick." Shall we work from the facts in sight and add others when they discover, as Newton did, or deductively from a theory whose practical application varies as the frequent disappointment which such application brings, as witness the many phases through which Lister has compulsorily passed his complicated antiseptic dressings, the last of which, moreover, adding a new element of danger—the risk of mercurial poisoning. It must be admittted that Tait's practice corroborates his logic as much as that of the antiseptics do theirs. Tait's end of the stick—he believes—is equally notched, if not more so. The whole question may still be *sub judice*, but of one thing we may be well assured, no amount of antiseptic precautions will compensate for bungling and loss of time in operating. In any event, here we stand on solid ground, and it is a lesson, yea! a revelation to see how

Tait in every conceivable item strives to avoid these errors. He constantly devises new ways, indeed, every appurtenance of the operation to be performed is ordered to economize time. The time and labor of the surgeon and the safety of the patient are thus husbanded, germs to the contrary, notwithstanding. Let me give an incomplete illustration: While the German operator at Berlin has not yet finished shaving and making antiseptic the parts in the operation of perineonhaphy, Tait's operation is well and completely done, and yet germs never interfere with the healing of the parts after the operation which Tait does on the perineum.

If you want to correct, or confirm your views of men and things as formed from what you read just "go abroad." Surely in things medical, "distance lends enchantment to the view" oftener than it does otherwise. This does not apply to Tait. His writings are a perfect reflex of what he thinks and does; but if you take the latter in the sense of substantial achievement the same cannot be said of Mr. John Clay, of Birmingham, with his asserted cure of cancer—Chian turpentine. Right here I am willing to retract the confidence I have elsewhere expressed in Mr. Clay,s writings on this ubject, for Chian turpentine, as a curative remedy for carcinoma, is a thing of the past; there are none so poor here, in the home of its origin, as to do it reverence with the probable exception of Mr. Clay himself. Chian turpentine has been exhaustively tried both in private and hospital practice in this kingdom and is proved, beyond all question, to be "a dead failure." At the Queen's Hospital, Birmingham, full opportunity was given for testing its virtures, Mr. Clay, himself, supplying the drug for the experiment. To employ a slang Americanism: "It is no good." Consequently, "It is unwept, unhonored and unsung." Cancer now as before the claims of Chian turpentine as an abortive means were heralded and persistently adhered to by Mr. Clay, is even more rampant with growing frequency and rebellion to treatment. "Chian turpentine may, as some other things do, slightly modify the course of cancer but cure it never!" So says an eminent surgeon of the Queen's Hospital who is familiar with all the phases of this subject. If there be any treatment for this dread malady, an early application of the knife is the only means offering any hope. And this reminds me of two operations cleverly done at the Queen's for carcinoma of the rectum, one of which I saw,—total ex-section of this portion of the gut; preceded in both cases by inguinal colotomy. This operation goes by the name of "Madelung's operation," and is now on trial as a preferable substitute for lumbar colotomy. The following histories of the cases have been kindly sent me by Mr. Marsh:

"I. C., æt. 29, admitted into the Queen's Hospital, Birmingham, England, February 23, 1889, with an adenoid carcinoma of the lower three and a half inches of rectum; involving the whole circumference except a small extent of the anterior wall. Duration of the disease about eighteen months. In Chicago, United States, seven months prior to admission, it was diagnosticated carcinoma, and for some months previously had been under treatment for "piles." March 1, 1889, the patient under an anæsthetic, the usual incision for inguinal colotomy was made and the colon found without difficulty; a loop of six inches was drawn down to anchor the upper end to guard against subsequent prolapse, and the bowel obliquely divided with the scissors. A few bleeding points were tied with fine catgut, and the edges of the lower opening were folded in and sutured with a continuous catgut suture (modified Lambert). This end was now returned to the abdominal cavity, and the the upper orifice which had been carefully held by an assistant, was sutured to the edges of the abdominal wound by interrupted sutures *passing through all the structures*. The man made an uninterrupted recovery, and was up on the tenth day. Ten days later, excision of the rectum was performed. An oval incision was made around the anus and deeply back to the coccyx with a scalpel, scissors being employed to complete the operation. A large cavity was left bounded by the sacrum behind, the bladder in front and the peritoneum above, the cavity of the latter not being opened, Hemorrhage was copious during the operation, but easily controlled by pressure subsequently, no vessel requiring ligature. The man again made a rapid recovery, and was discharged from the hospital on May 26, 1889, a small sinus remaining. A few months later this had quite closed, he felt well and strong, having gained over twenty pounds in weight, and able to walk easily ten to fifteen miles. He had control over his motions, and experienced no trouble from the artificial anus.

"August, 1890. He keeps strong and well, no sign of recurrence. For the last twelve months he has been driving a Hansom cab. Although he had been provided with an elastic belt with india rubber pad to fit the artificial anus, he had neglected to wear it, feeling more comfortable with a pad kept in place with a loose linen band. Consequently, there was a bulging of the abdominal wall and considerable prolapse of the bowel, notwithstanding that the mesentery had been rendered taut at the time of operation. The original belt, however, entirely remedies this, and since he has recommenced wearing it there has only been a slight prolapse. The perineal wound keeps soundly cicatrised, the tuberosities of the ischium appearing very prominent."

This case was exhibited at the annual meeting of the British Medical Association at Leeds, in 1889, and again at the annual meeting in Birmingham, 1890, and was favorably commented upon by those who saw it.

"Mrs. N. aet. 53. Duration of symptoms quite twelve months. Colotomy performed as described in previous cases on August 17, 1890. Did very well locally, but kept feeble constitutionally; very little recuperative power, and suffered a good deal of pain of growth which involved sphincter area and anal margin. Excision of rectum (four inches) on September 14. Lymphatics outside of rectum involved posteriorily some distance upwards in hollow of sacrum. Excision of these doubled length of operation and gravity. Patient rallied fairly well from primary shock, the packing removed following day, no hemorrhage, but good deal of serous oozing. Relapsed and gradually sank and died three days later—September 7, 1 p. m."

Mr. Marsh is a competent and experienced surgeon, with a healthy admixture of caution, self-possession and determination in operating. He is a disciple of Lister, and very soon will have the latest antiseptic dressing of the latter in full working operation at the Queen's Hospital, to which he is one of the attending surgeons. In answer to my question: "How do you account for Tait's success without the use of antiseptics," he said:

"Tait is a thorough aseptic operator." He is also the best abdominal surgeon in the world."

The Queen's Hospital is one of the best constructed, best regulated and best equipped institution I have ever seen, but one of its surgeons, a colleague of Mr. Marsh—Mr. Jordan Lloyd—has promised to soon show the "second best hospital in the world"—the Workhouse Infirmary of Birmingham. *Nous Verrons.*

Fairness in dealing with medical questions compels me to make another retraction. In my editorials in the Texas Courier-Record of Medicine, I pleaded for more respect to be shown the protest of Wharton Jones against the acceptance by the profession of Conheim's teaching concerning the immigation of the white blood corpuscles in inflammation.

On investigating the matter here, I am informed that:—"We never took Wharton Jones seriously," and that: "No fact in pathology seems to be fixed on a more stable foundation than Conheim's demonstration." Now let my good friend Ward, of Waxahachie, exultingly say: "I told you so." I can only rejoice that I make no claims to microscopy, and only knew Jones from his plausible writings. At Birmingham, however, I can estimate him at shorter range as I can others of whom I had heard much, but of whose "authority" hereafter, I shall, myself, not think "seriously." Truly yours,

HENRY K. LEAKE, M. D.

Birmingham, England, Sept. 6, 1890.

